# Natural diterpenes from coffee, cafestol and kahweol induce apoptosis through regulation of specificity protein 1 expression in human malignant pleural mesothelioma

**DOI:** 10.1186/1423-0127-19-60

**Published:** 2012-06-26

**Authors:** Kyung-Ae Lee, Jung-Il Chae, Jung-Hyun Shim

**Affiliations:** 1 Department of Biochemistry, College of Medicine, Soonchunhyang University, Ssangyong-dong, Seobuk-gu, Cheonan, Choongnam, 331-090, Republic of Korea; 2 Department of Dental Pharmacology, School of Dentistry, Brain Korea 21 Project, Chonbuk National University, Jeonju, 561-756, Republic of Korea

**Keywords:** Coffee, Cafestol, Kahweol, Apoptosis, Sp1, Human malignant pleural mesothelioma

## Abstract

**Background:**

Malignant pleural mesothelioma (MPM) is a highly aggressive cancer with a very poor prognosis. Several clinical studies such as immunotherapy, gene therapy and molecular targeting agents have been tried for treatment of malignant mesothelioma, however, there is no application for effective clinical treatment. Coffee has various biological functions such as anti-oxidant, anti-inflammatory, anti-mutagenic and anti-carcinogenic activities. The therapeutic activities of the bioactive compounds in coffee was sugested to influence intracellular signaling of MPM. Regarding to the cancer-related functions, In this study, suppression of Sp1 protein level followed by induction of MSTO-211H cell apoptosis by cafestol and kahweol were investigated in oreder to determine Sp1's potential as a significant target for human MPM therapy as well.

**Methods:**

Cells were treated separately with final concentration of cafestol and kahweol and the results were analyzed by MTS assay, DAPI staining, PI staining, luciferase assay, RT-PCR, and immunoblotting.

**Results:**

Viability of MSTO-211H and H28 cells were decreased, and apoptotic cell death was increased in MSTO-211H as a result of cafestol and kahweol treatment. Cafestol and kahweol increased Sub-G_1_ population and nuclear condensation in MSTO-211H cells. Roles of Sp1 in cell proliferation and apoptosis of the MSTO-211H cells by the Sp1 inhibitor of Mithramycin A were previously confirmed. Cafestol and kahweol significantly suppressed Sp1 protein levels. Kahweol slightly attenuated Sp1 mRNA, while Cafestol did not affect in MSTO-211H cells. Cafestol and kahweol modulated the promoter activity and protein expression level of the Sp1 regulatory genes including Cyclin D1, Mcl-1, and Survivin in mesothelioma cells. Apoptosis signaling cascade was activated by cleavages of Bid, Caspase-3, and PARP with cafestol and by upregulation of Bax, and downregulation of Bcl-_xl_ by kahweol.

**Conclusions:**

Sp1 can be a novel molecular target of cafestol and kahweol in human MPM.

## Background

Cancer is one of the leading causes of death in the world. Malignant pleural mesothelioma (MPM), the most common primary tumor of the pleura, is a highly aggressive cancer with a very poor prognosis. Early detection of MPM is difficult because of a lack of available tests able to identify MPM in advance [1,2]. Approximately 80% of MPM has been associated with respiratory exposure to asbestos fibers [3]. Because of the long latent period, the disease is expected to peak within 20–40 years after the exposure of asbestos [2]. Survival time of most patients is very short (median survival of 6–18 months) except in cases of complete resection [4]. Several clinical trials including conventional chemotherapy or radiotherapy were suggested as a treatment option for MPM, but they did not present successful therapeutic effectiveness [1]. Further understanding in mechanisms of mesothelial carcinogenesis is heavily related to development of novel treatments against biological pathways especially involved in proliferation, cell survival, and angiogenesis in other cancers [5]. However, no effective clinical application has been discovered regarding to it yet [6].

**Figure 1:**
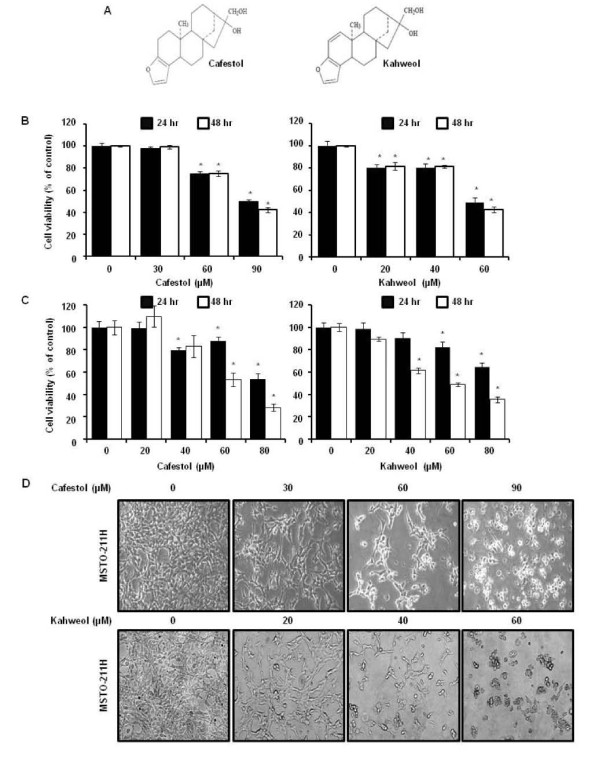
**The effect of cafestol and kahweol on cell viability in MSTO-211H cells.** (**A**) Chemical structure of cafestol and kahweol. The cell viability effect of cafestol and kahweol on MSTO-211H (**B**) or H28 (**C**) cells. MSTO-211H cells or H28 were treated with cafestol and kahweol in FBS-RPMI 1640 for various time. The viability of cells were determined with the MTS assay kit, as described under “Material and Methods”. Results are indicated as cell viability relative to untreated controls with cafestol and kahweol, as determined from three independent experiments. Data are represented as the mean ± SD. The asterisk indicated a significant difference compared with the negative control (*p* < 0.05). (**D**) The cell morphological changes of MSTO-211H cells treated or untreated with cafestol and kahweol for 48 hours.

Coffee is the most popular beverage in the world after water, and total transaction regarding to coffee consumption is equal to 10 billion US dollar worldwide. Coffee has various biological functions regarding to its chemopreventive potential such as anti-oxidant, anti-inflammatory, anti-mutagenic and anti-carcinogenic activities [7]. Several researches proved that a daily intake of 2–3 cups of coffee can be effective against coronary heart diseases, diabetes mellitus, cancer lines, Parkinson’s, and Alzheimer’s disease as well. Coffee is a complex mixture of a thousand chemicals containing potential bioactive molecules such as chlorogenic acid, caffeine, and two diterpenes including cafestol and kahweol [8]. The typical bean of *Coffea arabica* contains cafestol and kahweol, a structural analogue of cafestol, with individual concentrations ranging from 0.1-7mg/ml in coffee [9,10]. Cafestol and kahweol are fat-soluble compounds known as diterpene molecules (Figure 1A), which are present in oil derived from coffee beans. Relatively high levels of cafestol and kahweol are present in unfiltered coffee drinks such as espresso, French press, boiled coffees or Turkish coffee/Greek coffee, while filtered coffee and instant coffee contain low levels of cafestol and kahweol [11,12]. The high diterpenes content, cafestol and kahweol, in coffee is inversely associated with reported risk of colorectal cancer [13]. However, the anti-tumor mechanisms and molecular targets of cafestol and kahweol are poorly understood, especially in human malignant pleural mesothelioma.

Specificity protein (Sp) is a transcription factor with an additional eight family member, and is ubiquitously expressed in all mammalian cells [14]. Sp1 was recently defined as the Sp/krűppel-like transcription factor [15] and has many cellular functions including regulation of cellular processes, growth, and metastasis of human tumors by regulating oncogenes, tumor suppressor genes, cell cycle control molecules, growth-related signal transductions, angiogenesis-related factors, as well as apoptosis [16]. Additionally, Sp1 is highly expressed in various cancers such as breast carcinoma, thyroid cancer, hepatocellular carcinoma, pancreatic cancer, colorectal cancer, gastric cancer, and lung cancer [17-20]. Regarding to its cancer-related functions, Sp1 has been suggested as a novel target for cancer therapy.

Therapeutic activities of the bioactive compounds in coffee are thought to influence the intracellular signaling of MPM, although the association between diterpenes and Sp1signaling has not yet been clarified. In order to verify its therapeutic potential, we investigated whether cafestol and kahweol regulated Sp1 target proteins can induce apoptosis by suppressing Sp1 protein level in MSTO-211H cells.

## Methods

### Chemicals

Cafestol, Cycloheximide (CHX), and Mithramycin A (Mith A) were purchased from Sigma-Aldrich (St. Louis, Missouri). Kahweol was obtained from Santa Cruz Biotechnology, Inc. (Santa Cruz, CA). The chemicals were dissolved in dimethyl sulfoxide (DMSO).

### Antibodies

The following antibodies were purchased: anti-Cyclin D1 (M-20), anti-Sp1 (1C6), anti-Caspase-3 (H-277), horseradish-peroxidase-conjugated anti-mouse IgG, and horseradish-peroxidase-conjugated anti-rabbit IgG (all from Santa Cruz Biotechnology, Inc.), anti-Poly ADP-ribose polymerase (PARP) (BD Biosciences, San Diego, California), anti-Mcl-1, anti-Survivin, anti-Bid, anti-Bax, anti-Bcl_xl_ (Cell Signaling, Danvers, Massachusetts), and anti-β-Actin (AC-74) (Sigma-Aldrich, Inc. St. Louis, Missouri).

### Cell culture

MSTO-211H and H28 cells, a type of human mesothelioma cells, were obtained from the American Tissue Culture Collection (Manassas, Virginia). The MSTO-211H cells and H28 were maintained in Hyclone RPMI-1640 containing 5% and 10% fetal bovine serum (FBS), respectively, and 100 U/ml each of penicillin and streptomycin (Thermo scientific, Logan, Utah) at 37°C in a humidified chamber with 5% CO_2_ and 95% air. The medium was changed every two days.

### MTS assay

The cell viability of MSTO-211H and H28 cells was accessed using the MTS (3-(4,5-dimethylthiazol–2-yl)-5-(3–carboxymethoxyphenyl)-2-(4-sulfophenyl)-2H–tetraz-olium) Assay Kit (Promega, Madison, Wisconsin), according to the manufacturer’s instruction. The MSTO-211H cells (3 × 10^3^ cells/100ul/well) or H28 (1.5 × 10^3^ cells/200 μl) were grown on a 96-well microtiter plate for 24 hours. Cafestol and kahweol were added directly to the culture media with no more than 0.1% as final concentration of DMSO. Cells were treated with various concentration of cafestol and kahweol for 24 hours and 48 hours. MTS cell proliferation assay reagent was added, and samples were incubated at 37°C in 5% CO2 for two hours. Absorbance was measured at 490 nm using GloMax-Multi Microplate Multimode Reader (Promega, Madison, Wisconsin), and the difference between the test and reference wavelength was calculated. The cell viability was calculated using an equation: (optical density ratio of cafestol or kahweol-treated sample/non-treated sample) × 100 (%).

### DAPI staining

Apoptosis of cafestol or kahweol-treated cells was observed using 4’-6-diamidino-2-phenylindole (DAPI) staining. Nuclear condensation and fragmentation were observed using nucleic acid staining with DAPI (Sigma-Aldrich, Inc. St. Louis, Missouri). The MSTO-211H cells treated for 48 hours with 0–90 μM cafestol and 0–60 μM kahweol were harvested by trypsinization, washed with cold Phosphate Buffered Saline (PBS), and fixed in 100% methanol at room temperature for 20 minutes. The cells were spread on slide, stained with DAPI solution (2 μg/ml), and observed through a FluoView confocal laser microscope (Fluoview FV10i, Olympus Corporation, Tokyo, Japan).

### Propidium iodide staining

After 48 hours of cafestol or kahweol treatment, the detached cells (floaters) were collected by centrifugation and combined with adherent cells. The cells were washed with cold PBS, fixed in 70% ice-cold ethanol overnight at −20°C, and treated with 150 μg/ml RNase A and 20 μg/ml propidium iodide (PI; Sigma-Aldrich, Inc. St. Louis, Missouri). The stained cells were analyzed, and distribution of the cells in different phases of cell cycle was calculated using flow cytometry with a MACSQuant Analyzer (Miltenyi Biotec GmbH, Bergisch Gladbach, Germany).

### Reverse transcription-polymerase chain reaction

Total RNA was extracted from cells using TRIzol® Reagent (Life Technologies, Carlsbad, California), and first strand cDNA was synthesized from 2 μg of RNA using reverse transcriptase with Oligo-(dT) primer according to instructions for HelixCript^TM^ 1^st^-strand cDNA synthesis kit (NanoHelix, Korea). cDNA was obtained by PCR using β-actin-specific and Sp1-specific primers as described below under following PCR conditions (25 cycles: 1 min at 95°C, 1 min at 60°C, and 1 min at 72°C). β-actin primers were: 5'-GTG-GGG-CGC-CCC-AGG-CAC-CA-3' (forward) and 5'-CTC-CTT-AAT-GTC-ACG-CAC-GAT-TTC-3' (reverse). Forward Sp1 primers were: ATG CCT AAT ATT CAG TAT CAA GTA; reverse primers were: CCC TGA GGT GAC AGG CTG TGA. The PCR products were analyzed using 2% agarose gel electrophoresis.

### Luciferase Assays

MSTO-211H cells (6 × 10^4^) were seeded into 24 well plates in triplicate for 24 hrs. Transient transfection was performed using a LipofectAMINE2000 reagent (Invitrogen, Carlsbad, CA, USA). The Survivin-269 promoter constructs was kindly provided by Dr Sung-Dae Cho (Chonbuk National University, Jeon-ju, Korea). The mcl-1 promoter was obtained from Addgene (Cambridge, MA, USA). Cells were transfected with 250 ng of cyclin D1 (−1745-luc), mcl-1 (−325), or Survivin (−269), and 20 ng of β-gal using LipofectAMINE2000 reagent (Invitrogen, Carlsbad, CA, USA) for 24 h [21]. 48 hrs after cafestol and kahweol treatment, whole cell lysates were harvested, and firefly luciferase and galactosidase activity were determined with a Promega luciferase assay kit (Madison, WI) according to the manufacturer’s manual. After PBS wash, passive lysis buffer (200 μl) was added, the cells were then incubated for 1 hr with gentle shaking. Cell lysates (100 μl each) were mixed with 100 μl luciferase assay reagent, and firefly luciferase light emission was measured by GloMax™ Multi + MicroplateMulti Reader (Promega). Subsequently, 50 μl of β-galactosidase substrate was added in order to normalize the firefly luciferase data.

### Western blotting

The cafestol or kahweol treated cells were cultured for 48 hours and washed twice with cold PBS, then the cells were lysed with PRO-PREP™ Protein Extraction Solution (iNtRON Biotechnology, Korea) containing 1 μg/ml aprotinin, 1 μg/ml leupeptin, and 1 mM PMSF. The extracted protein was prepared in 200 μl of the extraction solution, containing about 1 × 10^7^ cells. The protein concentration was measured using DC Protein Assay Reagent (BIO-RAD Laboratories Inc., Hercules, California). Fifty micrograms of total cellular protein per lane were separated with 10 and 15% SDS-polyacrylamide gel electrophoresis and transferred onto polyvinylidene difluoride (PVDF) membranes. The membrane was blocked for two hours at room temperature with 5% non-fat dried milk in PBS containing 0.1% tween-20, and incubated with specific antibodies overnight at 4°C. The secondary antibodies to IgG conjugated with horseradish peroxidase were used, and the membrane was developed with the Pierce ECL Western Blotting Substrate (Thermo scientific, Rockford, Illinois) according to the manufacturer’s instructions.

### Statistical analysis

Data were presented as the mean ± SD from three independent experiments. Data analysis for statistical significance was done using Student’s *t*-test. Compared to the vehicle control, *p* value was less than 0.05, indicating statistical significance of the data.

## Results

### Cafestol and kahweol suppress the viability of mesothelioma cells

To determine the cell viability following treatment with cafestol and kahweol- 0j, we confirmed the growth inhibitory potential of MSTO-211H and H28 cells using the MTS assay. The results indicated that MSTO-211H and H28 cell viability for 24 and 48 hours was decreased in cafestol and kahweol dose-dependent as well as time-dependent manner (Figure 1B). The IC_50_ value of the cafestol and kahweol after 48 hrs of incubation was estimated as 82.07 μM and 56.00 μM in MSTO-211H cells and H28 cells, respectively. Cell viabilities were calculated as 99.3 ± 1.8%, 75.1 ± 2.6%, and 42.1 ± 2.2% of the control at 30, 60, and 90 μM cafestol in MSTO-211H cells and H28 cells, respectively. Kahweol treated cells had measured viabilities were 81.7 ± 3.4%, 81.3 ± 1.4%, and 42.6 ± 2.6% at 20, 40, and 60 μM. The IC_50_ of cafestol and kahweol for 48hr treatment in the H28 cells were calculated approximately 62.7 and 57.8 μg/ml (Figure 1C).

To evaluate the potential cell morphological changes as a result of treatment with cafestol and kahweol in malignant mesothelioma cells, MSTO-211H cells were treated with cafestol (0–90 μM) or kahweol (0–60 μM) of various concentrations and monitored for 48 hrs. The MSTO-211H cells was changed to round cell shape, cell shrinkage was observed, and cell volume was decreased in those treated with cafestol (60 and 90 μM) and kahweol (40 and 60 μM) (Figure 1C).

### Cafestol and kahweol treatments induce apoptotic cell death in MSTO-211H cells

**Figure 2:**
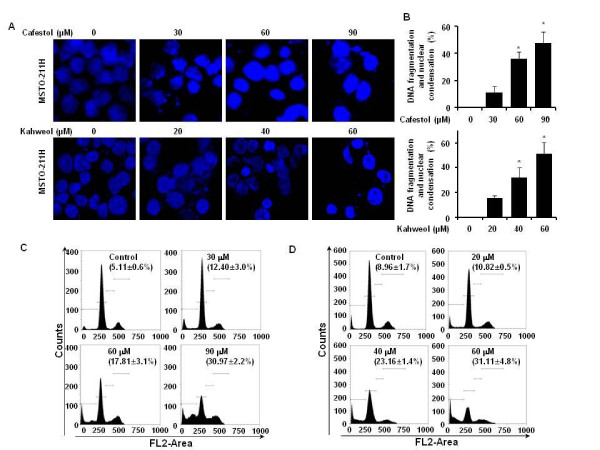
**The apoptotic effect induced by cafestol and kahweol in MSTO-211H cells.** Cells were incubated with cafestol (30, 60, and 90 μM) and kahweol (20, 40, and 60 μM) and untreated (DMSO) for 48 hours. The cells were harvested and prepared for DAPI staining and PI staining as described in the Methods section. (**A**) Analysis of DNA fragmentation and nuclear condensation by fluorescence microscopy (magnification X600) after cafestol and kahweol treatement in MSTO-211H cells. (**B**) DNA fragmentation and nuclear condensation were quantified, and the results in triplicates are expressed as the mean ± SD. Analysis of cell cycle by flow cytometry after cafestol (**C**) and kahweol (**D**) treatment of cells for 48 hours. Representative of Sub-G_1_ population. The cafestol or kahweol-treated cells were compared with untreated cells, and data are shown as the average of triplicate samples from three independent experiments. The asterisks (*) indicates *p* < 0.05 versus control cells.

The effect of cafestol and kahweol treatment on initiation of apoptosis in MSTO-211H cells was determined by nuclear morphology using DAPI staining, which allows visualization of nuclear shrinkage and fragmentation. Cafestol and kahweol treatment of mesothelioma cells led to an increase in nuclear condensation and fragmentation when compared to control group (Figure 2A, B). In order to evaluate whether the Sub-G_1_ population induction by cafestol and kahweol is related to apoptosis, cafestol and kahweol treated cells were marked with PI staining. When MSTO-211H cells were treated with cafestol and kahweol, increased number of cells in the Sub-G_1_ population was observed in 12 - 30% of cafestol-treated MSTO-211H cells and 10 - 31% of kahweol-treated MSTO-211H cells (Figure 2C, D).

### Cafestol and kahweol suppress specificity protein 1 protein in MSTO-211H cells

**Figure 3:**
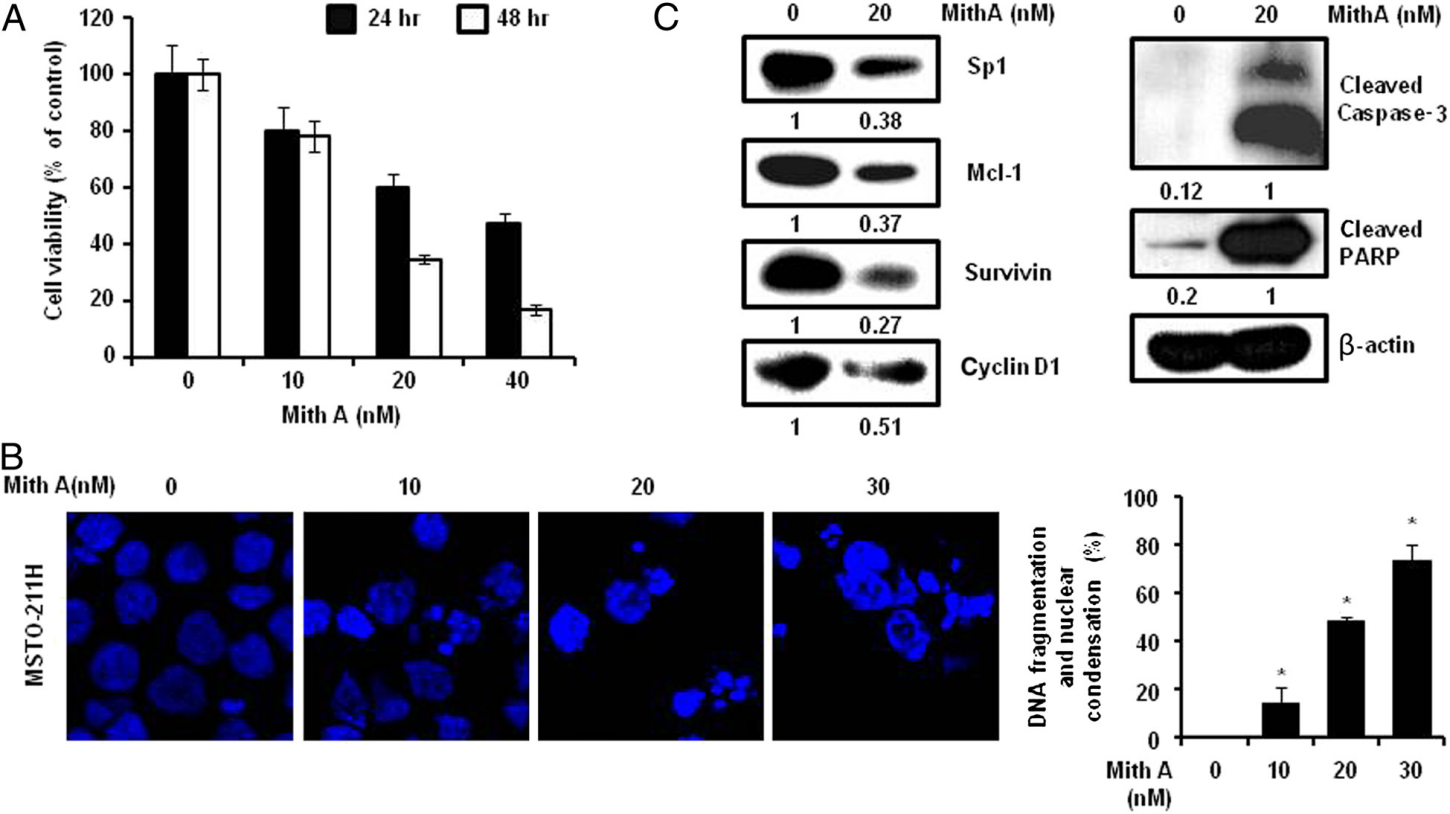
**Function of specificity protein 1 on cell viability and protein expression of MSTO-211H cells.** Sp1 expression is inhibited by mithramycin A (Mith A), a Sp1 regulatory inhibitor. (**A**) The cell viability effects of mithrramycin on MSTO-211H cells. MSTO-211H cells (3 × 10^3^ cells/200 μl) were treated with Mith A of various concentrations (10–40 μM) in 5% FBS-RPMI 1640 for 24 and 48 hours. The cell proliferation effects of Mith A-treated MSTO-211H cells was determined by the MTS assay. (**B**) Analysis of DNA fragmentation and nuclear condensation by fluorescence microscopy (magnification X600) after Mith A treatment in MSTO-211H cells. DNA fragmentation and nuclear condensation were quantified, and the results in triplicate are expressed as the mean ± SD. Data are indicated from three independent experiments. The asterisks (*) indicates *p* < 0.05 versus control cells. (**C**) The effect of Mith A on the expression of Sp1 protein, Sp1 regulatory proteins and apoptotic proteins in MSTO-211H cells. MSTO-211H cells were treated with Mith A of 20 μM for 48 hours. The effect of Mith A on Sp1 protein expression levels was determined by immunoblotting, as described in the Materials and Methods.

**Figure 4:**
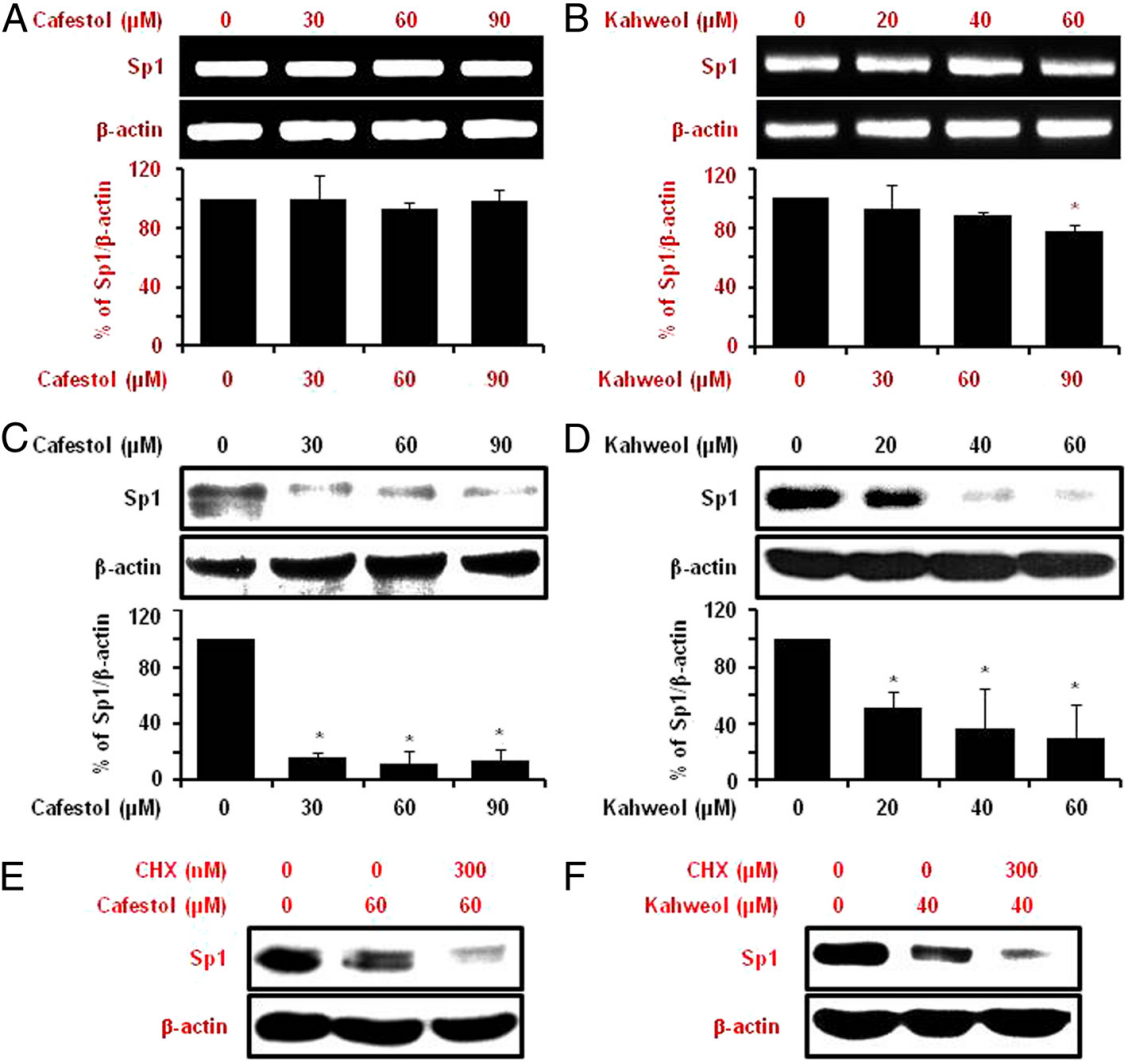
**The effect of cafestol and kahweol on Sp1 mRNA and protein expression in MSTO-211H cells.** The effect of cafestol (**A**, 0–90 μM) and kahweol (**B**, 0–60 μM) for 48 hours on Sp1 mRNA expression was determined by RT-PCR. The graphs indicate the ratio of Sp1 to β-actin expression. The effect of cafestol (**C**) and kahweol (**D**) for 48 hours on Sp1 protein expression was determined by western blotting. β-actin was probed to determine the equal loading of protein extract from each treatment. The graphs indicate the ratio of Sp1 to β-actin or Actin expression. Results are shown as the mean ± SD of three independent experiments. The asterisks (*, *P* < 0.05) indicated a significant change relative to cafestol or kahweol-treated cells compared with untreated cells. The effect of cafestol (**E**) and kahweol (**F**) on Sp1 protein turnover in MSTO-211H cells. The protein lysates were obtained from cells pretreated with protein synthesis inhibitor such as cyclohexmide (CHX) for 1 hour and then exposed to cafestol (E) and kahweol (F) for 48 hours. The protein expression of Sp1 were analyzed by western blot analysis.

**Figure 5:**
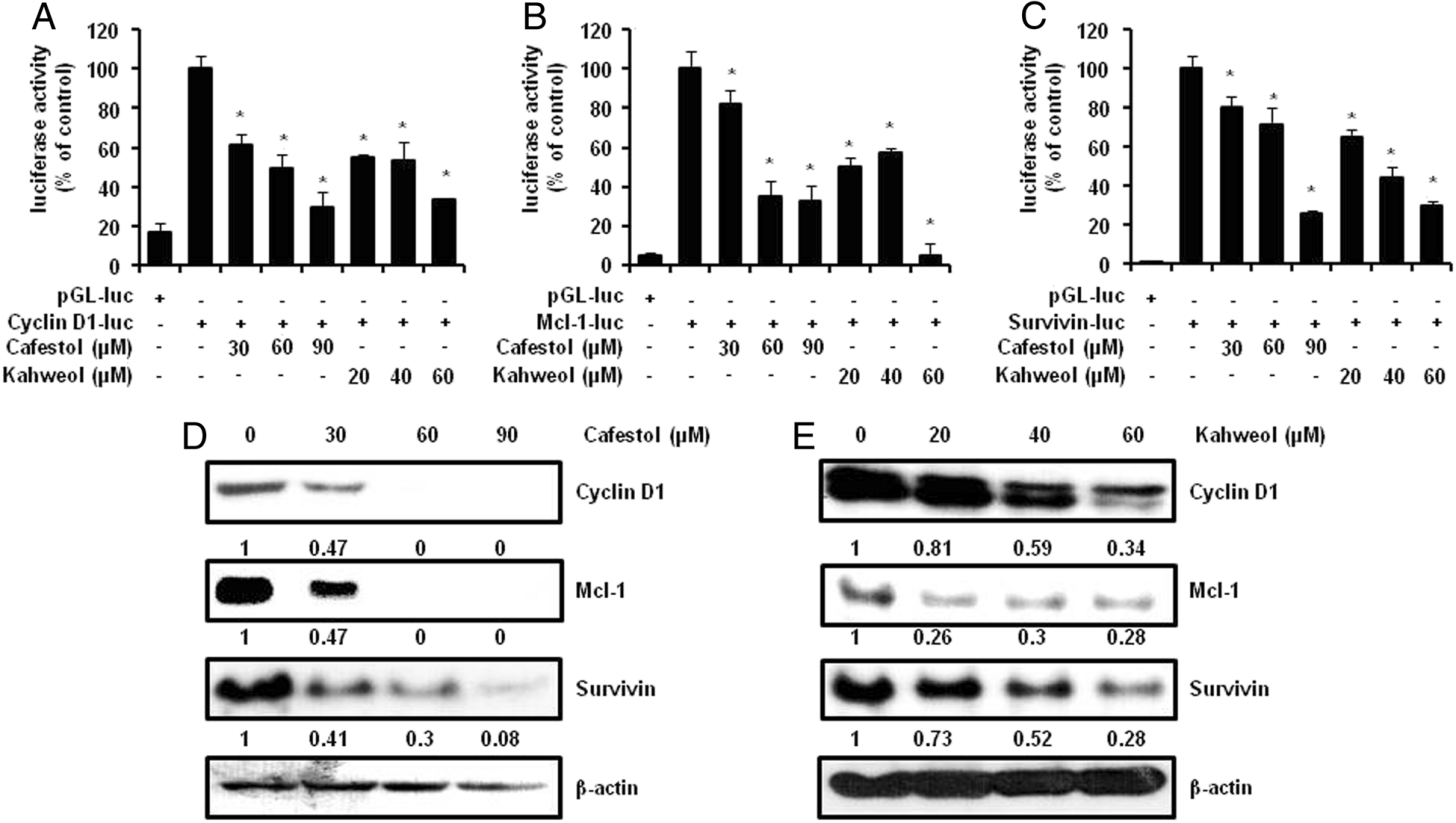
**The effect of cafestol and kahweol on downstream target proteins by the specificity protein 1 (Sp1).** The firefly luciferase Cyclin D1(**A**), Mcl-1 (**B**), or Survivin (**C**) reporter gene activity was assessed. For the reporter gene assay, MSTO-211H was transfected with a plasmid mixture containing the Cyclin D1, mcl-1, or survivin luciferase reporter gene (250 ng) and the β-gal gene (20 ng) for normalization. At 24 hours after transfection, cells were treated with various concentrations of cafestol or kaweol for 48 hours. Results are shown as the mean ± SD of triplicate samples from three independent experiments. The asterisks (*, *P* < 0.05) indicated a significant change relative to cafestol or kahweol-treated cells compared with untreated cells. The effect of cafestol (**D**) and kahweol (**E**) for 48 hours on Cyclin D1, Mcl-1, and Survivin was determined by Western blots. Fifty micrograms of cellular extract per lane were separated on the SDS-PAGE gel as described in materials and methods. Equal loading of protein was confirmed by incubating the same membrane with an anti-β-Actin antibody.

Numerous studies have documented that the levels of transcription factor Sp1 expression dramatically increased during transformation, indicating a critical influence in tumor development or maintenance. Transcriptional response targeting genes containing Sp1 binding site in their promoters are involved in many cellular functions ranging from differentiation to cell cycle progression, proliferation and apoptosis [22]. In order to confirm the Sp1 influence on MSTO-211H cell proliferation, MSTO-211H cells were treated with Mith A, a known Sp1 inhibitor for 24 and 48 hours. The cells treated with 10–40 μM Mith A demonstrated decrease in cell viability (Figure 3A) and induction of nuclear condensation and fragmentation (Figure 3B). Sp1 expression and the expression of Sp1 regulatory proteins (Cyclin D1, Mcl-1, and Survivin) were significantly downregulated by Mith A as well. In addition, Apoptosis-related proteins including caspase-3 and PARP were activated by Mith A (Figure 3C). Regarding to interaction with coffee, both cafestol and kahweol significantly suppressed Sp1 protein (Figure 4C, D). However, cafestol did not suppressed Sp1 mRNA on MSTO-211H cells (Figure 4A), and Kahweol slightly inhibited Sp1 mRNA (Figure 4B). When CHX-pretreated MSTO-211H cells were incubated with cafestol and kahweol, degradation of Sp1 protein by cafestol and kahweol was additionally enhanced (Figure 4E, F). Figure 5A, B, and C showed that cafestol and kahweol clearly attenuated Cyclin D1, Mcl-1, and Survivin promoter activities. Cafestol and kahweol also significantly suppressed Cyclin D1, Mcl-1, and Survivin protein levels of expression (Figure 5D, E).

### Cafestol and kahweol regulate the expression of anti-apoptotic and apoptotic molecules in MSTO-211H cells

**Figure 6:**
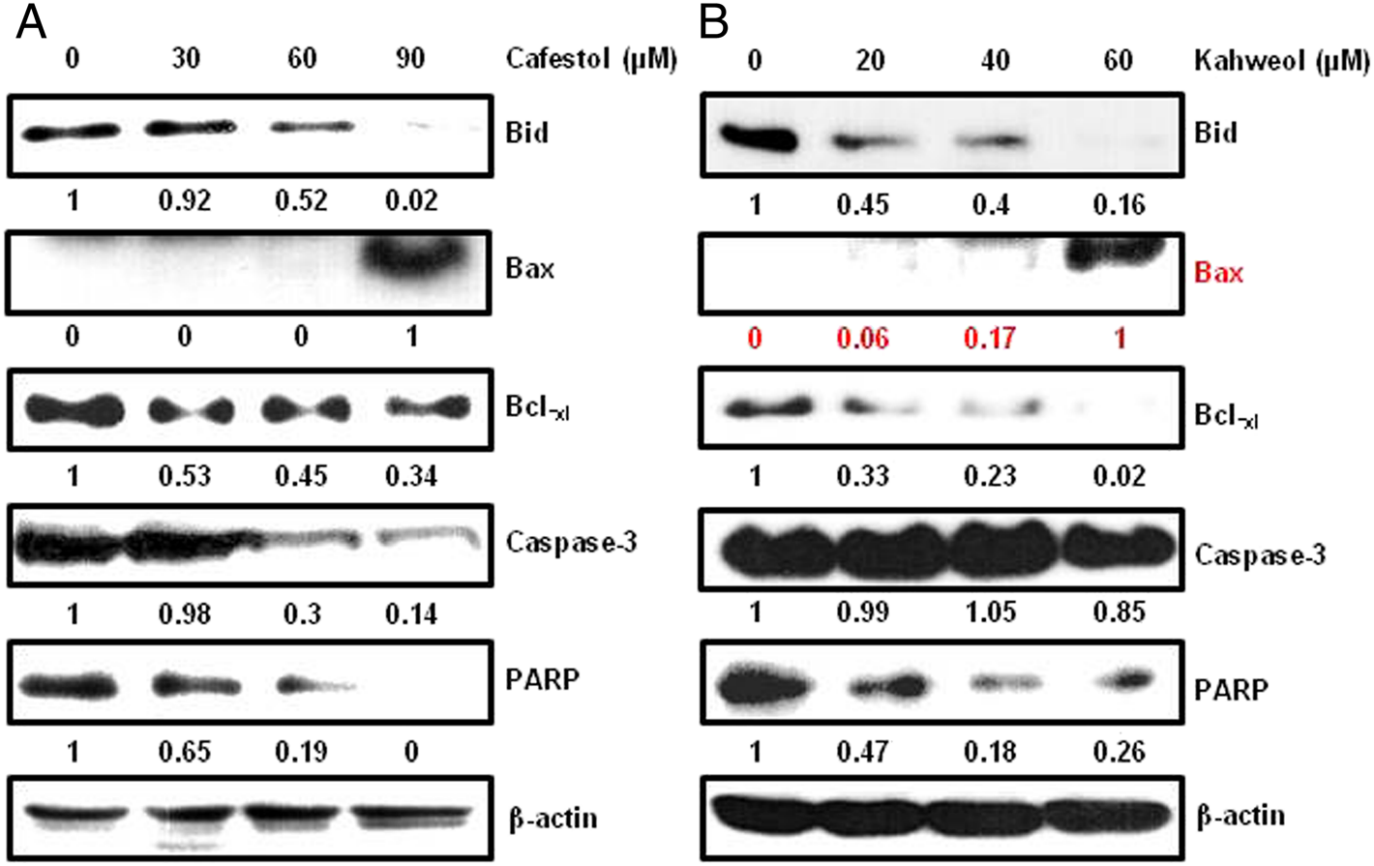
**The effect of cafestol and kahweol on apoptosis of MSTO-211H cells.** Fifty micrograms of cellular extract per lane were separated on an SDS-PAGE gel as described in materials and methods. Immunoblot detection of Bid, Caspase-3, and PARP, upregulation of Bax, downregulation of Bcl-_xl_ in whole cell lysates from various concentration of cafestol and kahweol-treated MSTO-211H cells for 48 hours. Equal loading of protein was confirmed by incubating the same membrane with anti-β-Actin antibody.

Treatment of cells with cafestol and kahweol regulates the expression levels of various apoptotic proteins. (Figure 6A, B). Immunoblotting technique was used to analyze levels of several pro- and anti-apoptotic proteins, to determine whether treatment with cafestol and kahweol regulates expression of apoptosis-related proteins in MSTO-211H cells. Activation of Bid, Caspase-3, and PARP, decreased Bcl-_xl_ and increased Bax in cafestol and kahweol-treated MSTO-211H cells. Inactivated form of PARP was decreased similarly to that of Sp1.

## Discussion

Coffee, in the form of roasted coffee beans, is one of the most widely consumed beverages worldwide. The diterpenes, cafestol and kahweol, were first identified as the specific lipidic fraction responsible for the chemopreventive effects of coffee in animal studies. Cafestol and kahweol have exhibited numerous beneficial health effects activities [23-26], however, chemoprotective mechanisms of cafestol and kahweol are not fully understood.

Prognostic determinants of MPM are non-epithelioid histological subtype, advanced stage, poor performance status, low hemoglobin level, leucocytosis, and thrombocytosis [27]. In MPM, multimodality treatment including radiation, surgery, and chemotherapy is used within a limited group of patients, and response rate of chemotherapy application for naïve patients is within 20-40% due to limited efficacy [28]. Various clinical trials which have included gene therapy and molecular targeting agents have consistently been done, although results from these studies have yet to be applied for clinical treatment [4,6].

Transcription factor Sp1 is known to be regulated by molecular target genes in various biological processes including differentiation, metabolism, cell growth, angiogenesis and apoptosis [14]. Sp1 is overexpressed in various human tumors, which may upregulate genes associated with tumor development, growth, and metastasis by binding onto promoter sequences [17-20]. Thus, Sp1 protein levels are expected to be a negative prognostic factor and potential therapeutic target for cancer chemotherapy [29]. Transcriptional factors, such as NF-κB and AP-1, have previously been identified as impending targets for therapy in patients with MPM [30,31]. However, the association between Sp1 and MPM has not yet been defined. In addition, the pharmacological effects of cafestol and kahweol and their involvement in the regulation of the transcriptional factors have been studied for cancer therapy [32-34], however, effects of cafestol and kahweol treatment on human mesothelioma cells have not been reported.

In this study, we focused on whether cafestol and kahweol suppressed mesothelioma cell growth (MSTO-211H and H28) and whether cafestol and kahweol can inhibit the expression of transcription factor Sp1 and induce apoptotic cell death in MSTO-211H cells. In previous studies, we confirmed the role of Sp1 in cell proliferation and apoptosis of MSTO-211H cells by RNA interference with small interfering Sp1. Our results confirmed that Mith A induced nuclear condensation and apoptosis of MSTO-211H cells and inhibited the expression of Sp1 and Sp1 regulatory proteins. Cafestol and kahweol inhibited cell viability and induced apoptotic cell death in MSTO-211H cells. Sp1 could contribute to cell growth via numerous gene expression regulation in various cancers [14]. Also, promoters of many apoptosis-related genes including Bcl-_xl_, survivin, Bax, and caspase-3 contain Sp1-binding sites [35]. Cafestol and kahweol inhibit Sp1 protein level and regulate Sp1 target proteins, such as Cyclin D1, Mcl-1, and Survivin. Regulation of Sp1 protein level was done via protein synthesis. We strongly suggest that cafestol and kahweol suppressed Sp1 protein expression and down-regulated Sp1-dependent gene expression. Cafestol and kahweol were also involved in apoptosis-related proteins.

Our results demonstrate Sp1 as an efficient therapeutic target of cancer. Sp1 expression levels increase during transformation, which can play a critical role in tumor development or maintenance. Although Sp1 deregulation is advantageous for treating tumor cells, it is reported that overexpression of Sp1 induces apoptosis of untransformed cells or cancer cells. Activation of apoptotic pathways by Sp1 overexpression is cell type-specific [18,36]. Despite elevation of Sp1, a decrease in Sp1 gene transcription level may also occur through indirect mechanisms by autoregulation of Sp1 [22]. Downregulation of Sp1 for cancer therapy is essential. Cafestol and kahweol treatment was shown to exhibit cancer therapeutic effects in MSTO-211H cells. However, definite verification of the inhibitory mechanisms of cafestol and kahweol at the molecular pathway of carcinogenesis is in demand. Cafestol and kahweol may have valuable chemopreventive effects in non-cancer patients as well as chemotherapeutic effects in cancer patients. Tumor necrosis factor-related apoptosis-inducing ligand (TRAIL) is known as a possible anti-cancer agent, but it may be inappropriate as a chemotherapeutic agent in isolation for cancer treatment because of drug resistance observed in some patients. A combination of kahweol and TRAIL induces apoptosis in various cancer cells [37]. This report might explain a role of kahweol as supplemental adjuvant and could present a novel strategy for cancer therapy. Heterogeneity in the study design of coffee regarding to gender could account for the difference in results observed [9]. As other experiments conducted in tumor cell-xenograft animal models, *in vivo* studies for MPM are needed to prove the safety and effectiveness for clinical applications. Cafestol and kahweol have similar functions, but they exhibit different degrees of bioactivity and stability [23,38]. For the most conventional approach of chemotherapies and chemoprevention of cancer, cafestol and kahweol need to be studied to determine minimum levels required to inhibit incidence of other diseases. Optimal ratio of cafestol and kahweol for individual for safe and efficient clinical application as dietary treatments needs to be determined as well. Water-soluble properties of cafestol and kahweol [7] may provide advantages for extraction and absorption in humans. For elimination of coffee constituents' side effects, it may be more effective to administer isolated cafestol and kahweol instead of increasing coffee consumption. Also, since most of consumed coffee is filtered, the idea of administering the extracted cafestol and kahweol can be very persuasive. Several studies do not have reported that excess amounts of coffee consumption above 6 cups/day influence the risk of cancer occurrence [9], however, the risk of other diseases might be increased by the coffee’s consumptions.

## Conclusions

MPM was influenced by the chemotherapeutic effects of cafestol and kahweol. We suggest that cafestol and kahweol regulate Sp1 target proteins, resulting in apoptosis by the suppression of Sp1 levels in MSTO-211H cells. Sp1 can be used as an effective therapeutic target in cancer research, and cafestol and kahweol are potential cancer drugs or adjuvants as chemotherapeutic agents for MPM.

## Abbreviations

MPM, Malignant pleural mesothelioma; Sp, Specificity protein; GST, Glutathione S transferase; DMSO, Dimethyl sulfoxide; Mith A, Mithramycin A; PARP, Poly ADP-ribose polymerase; FBS, Fetal bovine serum; MTS, MTS (3-(4,5-dimethylthiazol–2-yl)-5-(3–carboxymethoxyphenyl)-2-(4-sulfophenyl)-2H–tetraz-olium); DAPI, 4’-6-diamidino-2-phenylindole; PI, Propidium iodide.

## Misc

Jung-Il Chae Kyung-Ae Lee and Jung-Il Chae contributed equally to this study.

## Competing interests

No potential conflicts of interest are declared.

## Authors’ contributions

J-HS designed research and wrote the manuscript. K-AL and J-IC carried out research experiments and analyzed data. All authors read and approved the final manuscript.
